# COVID-19 Vaccination Acceptance among Healthcare Workers and General Population at the Very Beginning of the National Vaccination Program in Poland: A Cross-Sectional, Exploratory Study

**DOI:** 10.3390/vaccines10010066

**Published:** 2021-12-31

**Authors:** Karolina Lindner-Pawłowicz, Agnieszka Mydlikowska-Śmigórska, Kamila Łampika, Małgorzata Sobieszczańska

**Affiliations:** 1 Clinical Department of Geriatrics, Wroclaw Medical University, 66 Skłodowskiej-Curie Str., 30-688 Wrocław, Poland; malgorzata.sobieszczanska@umw.edu.pl; 2 Alzheimer’s Center Ltd., 12 Jana Pawła II Str., 59-330 Ścinawa, Poland; a.mydlikowska@gmail.com; 3 Department of Medical Humanities and Social Science, Wrocław Medical University, 7 Mikulicza-Radeckiego Str., 50-368 Wrocław, Poland; kamila.lampika@umw.edu.pl

**Keywords:** COVID-19, vaccination, hesitancy, population, healthcare workers

## Abstract

The aim of the study was to assess the acceptance level of COVID-19 vaccination among healthcare workers (HCW) and the general population in Poland at the start of the national COVID-19 vaccination program from 18–31 December 2020. A cross-sectional anonymous survey was conducted in a group of 1976 people: 1042 health professionals and 934 non-medical professionals using an on-line questionnaire. The most skeptical about the COVID-19 vaccine were students of non-medical faculties, non-medical professions, and administrative–technical health service staff (26.2%, 38.7% and 41.2%, respectively). The most positive attitude to vaccination was reported by doctors, medical students and pharmacists (80.6%, 76.9% and 65.7%, respectively). Doctors (64.7%) and medical students (63.7%) most often declared confidence in vaccines compared to nurses (34.5%). Distrust about vaccine safety was declared by nurses (46.6%) and pharmacists (40.0%). HCW encouraged others to vaccinate more eagerly (65.8%) than non-medical professions (28.3%). Thus, a considerable proportion of HCW in Poland expressed concern about vaccines just prior to the beginning of the COVID-19 immunization program. The significant decrease in the willingness to vaccinate observed in Poland towards the end of 2021 must be considered in the light of the serious COVID-19 vaccination hesitancy in the Polish population.

## 1. Introduction

Creating COVID-19 vaccines within less than 1 year of the outbreak of the pandemic seems to be one of the most spectacular technological achievements in medicine [1]. However, not only in Poland, vivid activity by anti-vaccination movements has been observed in recent years, which is particularly intense on the internet and social media [2]. Vaccination hesitancy is related to the acceptance delay or even refusal of vaccination despite the availability of vaccines [3,4]. Especially disturbing is that vaccination hesitancy in Poland is also observed among healthcare workers [5,6].

Similar problems were observed in other countries, where hesitancy concerned both the general public and a certain percentage of the health service [7,8].

Data from a global survey of potential acceptance of a COVID-19 vaccine published in October 2020 presented results from 19 countries. The question ‘If a COVID-19 vaccine is proven safe and effective and is available, I will take it’ was answered positively by: 88.62% of respondents from China, 85.36% from Brazil, 81.58% from South Africa, 79.79% from South Korea, 76.25% from Mexico, 75.42% from the US, 74.53% from India, 74.33% from Spain, 71.93% from Ecuador, 71.48% from Great Britain, 70.79% from Italy, 66.74% from Canada, 68.42% from Germany, 67.94% from Singapore, 65.23% from Sweden, 65.22% from Nigeria, 58.89% from France, 56.31% from Poland, and 54.85% from Russia. Polish society was one of the most skeptical of the hypothetical vaccine against COVID-19. Importantly, Poland had the highest percentage of negative responses among the surveyed countries (27.3%) [9].

At the time when COVID-19 vaccine production was being realized, many anti-vaccine theories and pseudoscientific theories, spreading mainly on the internet, were observed in public space in Poland, which increased fears of various vaccines, including that against COVID-19, and the percentage of Poles declaring their willingness to take such a vaccine was permanently low [10]. Anti-vaccine attitudes were strengthened, e.g., by the open letter ‘Appeal of scientists and doctors regarding vaccination against the SARS-CoV-2 coronavirus’ dated 20 November 2020, in which a group of representatives of the scientific and medical community raised serious doubts about the safety of anti-COVID-19 vaccines [11].

When the start of the national COVID-19 vaccination program in Poland was announced, there was, unfortunately, a lack of a strong promotion campaign, during which doctors and scientists could have presented the current state of knowledge about the new vaccine [12]. It is known that in all societies healthcare professionals are perceived as the opinion-forming leaders in the health field, therefore they could certainly elucidate many questionable issues regarding vaccination against COVID-19 [13].

At the beginning of the national COVID-19 vaccination program in Poland, it seemed interesting to study attitudes about the new available vaccines of both healthcare professionals, the group that started vaccination first and should promote the vaccination action, and also people not working in the health care sector.

## 2. Materials and Methods

### 2.1. Design and Study Collection

The study was performed by means of the online anonymous survey prepared as a Google Form that was disseminated through social media as well by sending links to the survey by email. The survey was carried out on 18–31 December 2020, i.e., just before and at the very onset of the national anti-COVID vaccination program in Poland that was launched on 27 December 2020.

The survey was addressed to persons with various levels of medical education, not only those routinely in contact with patients, like physicians, nurses, rescuers, physiotherapists, and psychologists, but also to medical students and administrative-technical staff of the health service. The group consisting of rescuers, physiotherapists, and psychologists was termed ‘other medical personnel’. On the other hand, we have questioned respondents not related professionally to health care, including non-medical students, and this group was named ‘non-medical professions’.

The study was performed with the help of the survey containing the questions conceived by the authors. The questions concerned basic demographic data and views of respondents on COVID-19 vaccination. The sociodemographic data collected comprised age, sex, education, professional status, occupation, size of the town/city in which a respondent lives—six questions in total. Attitudes towards COVID-19 vaccination were tested using questions about the willingness to undergo vaccination, opinions concerning the safety of COVID-19 vaccine, sharing the respondents’ views with other people on vaccination and opinions concerning the obligation to be vaccinated against COVID-19.

### 2.2. Statistical Methods

For purposes of the univariate analysis of categorical variables, a chi-square test with the post-hoc Bonferroni adjustment was used. Like the post-hoc tests used in the context of ANOVA, this adjustment is used to counteract the errors that can occur when multiple comparisons are made. When required, Yates’ correction for continuity was also applied. In addition, Cramer’s V was employed to evaluate the strength of associations between the pairs of the analyzed variables.

## 3. Results

### 3.1. Study Population

**Figure 1:**
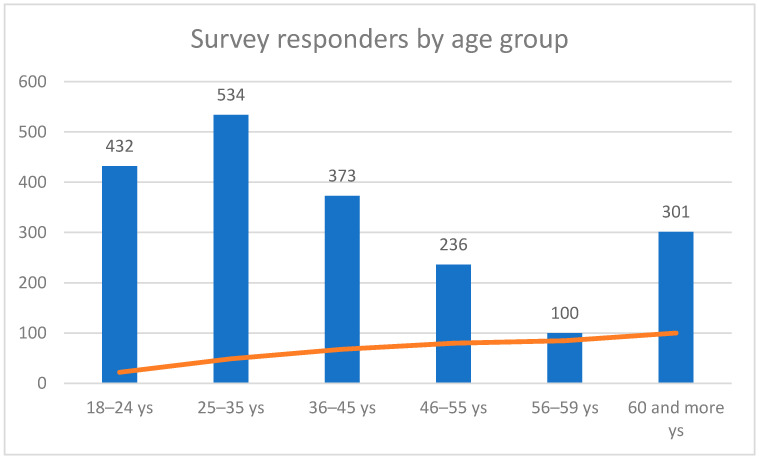
Quantity of the individual age groups in the examined population.

**Figure 2:**
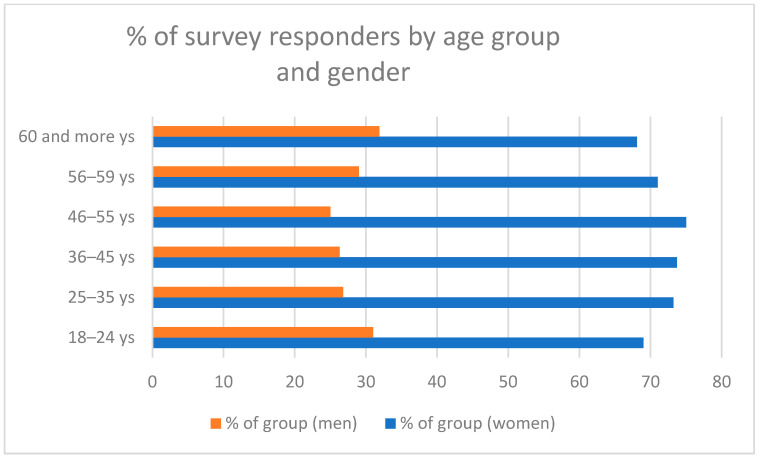
Survey respondents by age and gender.

**Figure 3:**
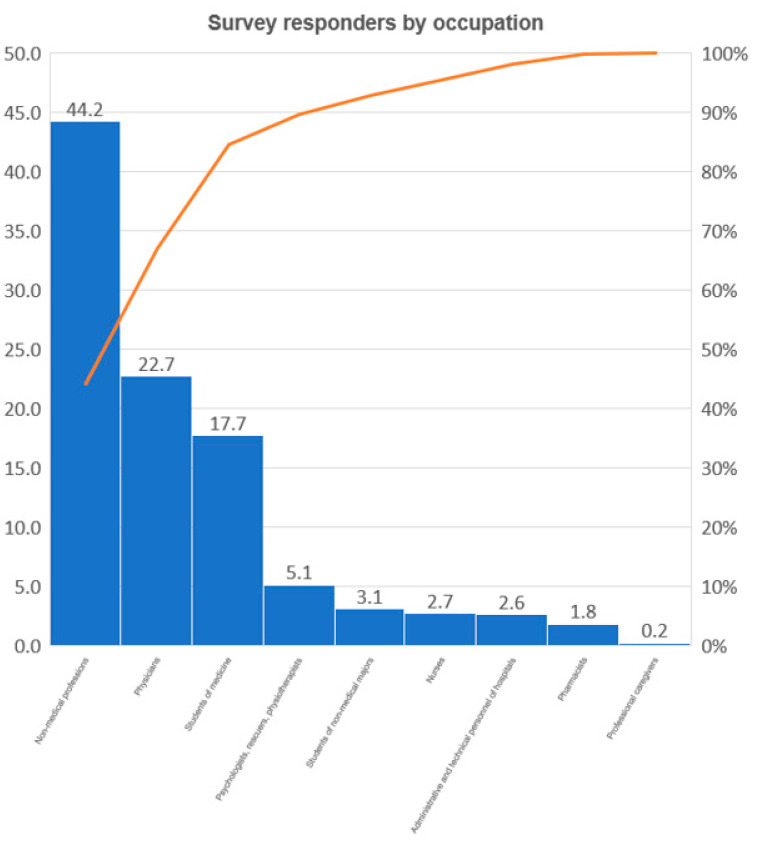
Survey respondents presented in professional group subsets.

The study included 1976 people ranging in age from 18 years to 94 years (median 39.55 years; SD ± 15.93 years), including 1042 health professionals and 934 people from the general population (non-medical professions). The exact numbers and characteristics of the individual subgroups of the respondents are presented in Figure 1, Figure 2 and Figure 3 and Table 1.

### 3.2. Profession and Attitude towards the Coronavirus Disease 2019 (COVID-19) Vaccination

Occupations performed by the surveyed respondents were associated with statistically significant differences in the approach to COVID-19 vaccination [χ^2^ (21,1976) = 335.147; *p <* 0.005; Cramer’s V = 0.238; *p* < 0.005]. Students of non-medical majors turned out to be the most skeptical of the idea of vaccination—only 26.2% of them declared they would undergo vaccination, while 19.7% did not intend to vaccinate. In the case of administrative and technical hospital staff, 41.2% were willing to undergo vaccination, and 17.6% expressed the opposite. Among the respondents outside medical professions, 38.7% declared they would undergo vaccination, and 19.2% did not intend to do so.

As for the decisions to accept the COVID-19 vaccine postponed until obtaining the additional information on the vaccine, i.e., a kind of vaccine or adverse effects noted in the other previously vaccinated persons, such assurances were made in the survey by as many as 54.1% of non-medical students and 41.2% of administrative and technical staff of hospitals, and 32.0% of non-medical professions.

In turn, the surveyed doctors appeared to be the most positive about COVID-19 vaccination. As many as 80.6% of them declared they would undergo vaccination and only 3.3% did not intend to take a vaccine. On the other hand, 76.9% of medical students surveyed would like to be vaccinated and 3.1% were not willing to do so. Among the pharmacists tested, 65.7% of them expressed a willingness to be vaccinated, while 5.7% were of the opposite opinion.

**Figure 4:**
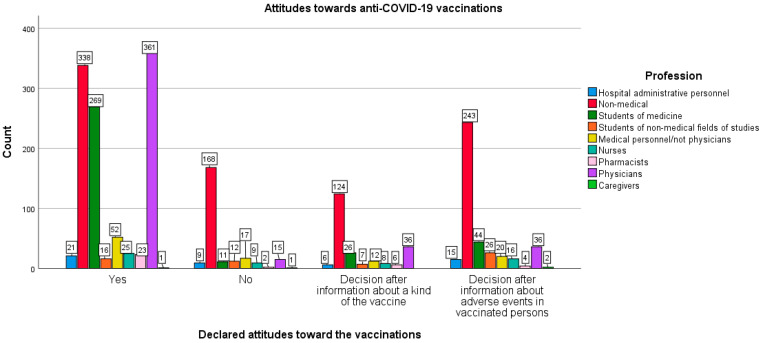
Declared attitude towards the coronavirus disease 2019 (COVID-19) vaccination in the particular profession groups.

Surprisingly, only 52.1% of other medical personnel (i.e., excluding doctors) and 43.1% of nurses declared their willingness to undergo vaccinations. The anti-vaccination rate in these two groups was 17.0% and 15.5%, respectively (see Figure 4).

### 3.3. Gender and Attitude towards the COVID-19 Vaccination

**Figure 5:**
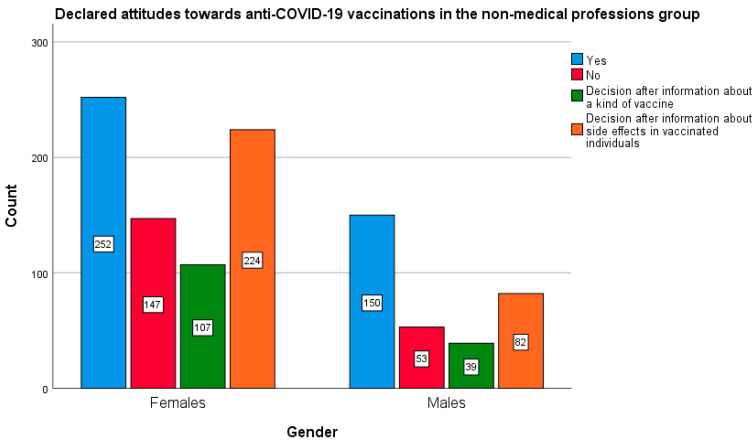
Gender and declared attitude towards the COVID-19 vaccination in the group of non-medical professions.

**Figure 6:**
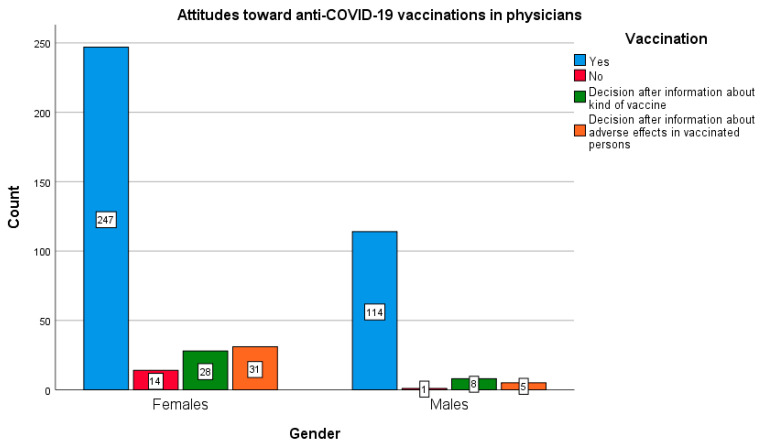
Gender and declared attitude towards COVID-19 vaccination in the group of doctors.

The in-depth statistical analysis showed that the gender of the respondents significantly differentiated the results of two professional groups: non-medical (χ^2^ (3873) = 12.176; *p* < 0.05; Cramer’s V = 0.118; *p* < 0.05) and doctors (χ^2^ (3448) = 9.641; *p* < 0.05; Cramer V = 0.147; *p* < 0.05). In both of the above-mentioned groups, men declared greater willingness to undergo vaccinations as compared with women (Figure 5 and Figure 6). This relationship was not found in the remaining study groups.

In the group of non-medical professions, the proportions between the sexes were, as follows: 34.8% of women declared a willingness to vaccinate against COVID-19 vs. 47.1% of men, while 20.3% of women and 16.9% of men did not intend to be vaccinated. As for the group of doctors surveyed, 77.2% of women wanted to be vaccinated against COVID-19, as compared to 89.1% of men, whereas only 4.4% of women and 0.8% of men declared that they would not be vaccinated against COVID-19.

### 3.4. Beliefs about Safety of the COVID-19 Vaccination

**Figure 7:**
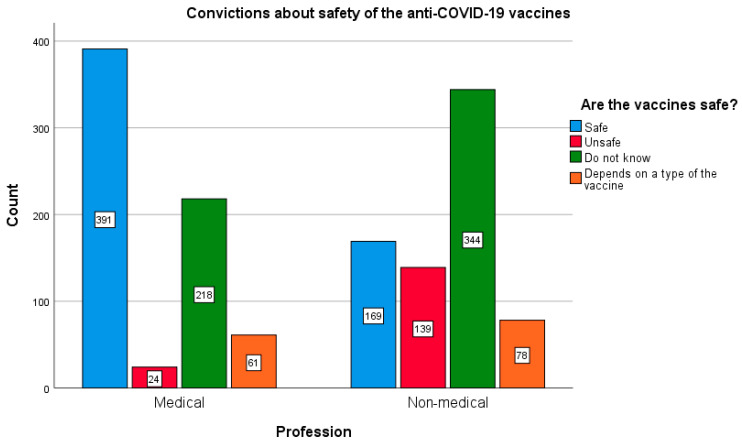
Beliefs about the safety of the COVID-19 vaccination among representatives of medical and non-medical professions.

In general, surveyed persons related to the medical care were more likely to trust the COVID-19 vaccine; 58.9% of respondents expressed a belief that the vaccine is safe, while in the group of non-medical professions, only 27% of respondents believed that the vaccine does not pose a health risk. At the same time, healthcare professionals raised doubts about the safety of the vaccine less frequently compared to respondents not professionally related to health care, 29.4% and 45.4%, respectively; χ^2^ (3.1424) = 254.674; *p* < 0.005. The magnitude of the above effect is moderate (Cramer’s V = 0.358; *p* < 0.005). This effect is maintained taking into account the gender of respondents from both groups (women: χ^2^ (3.1424) = 198.687; *p* < 0.005; Cramer’s V = 0.374; *p* < 0.005, and men: χ^2^ (3.565) = 66.774; *p* < 0.005; Cramer’s V = 0.344; *p* < 0.005). Differences in beliefs about the safety of vaccination against COVID-19 among representatives of medical and non-medical professions are depicted in Figure 7.

Analyzing the aforementioned aspect in more detail, it was found that in the medical group, doctors (64.7%) and medical students (63.7%) most often declared confidence in the COVID-19 vaccines. The remaining persons related to health care declared confidence in those vaccines much less frequently, i.e., 40.0% of pharmacists, 34.5% of nurses and 23.8% of other medical staff.

**Figure 8:**
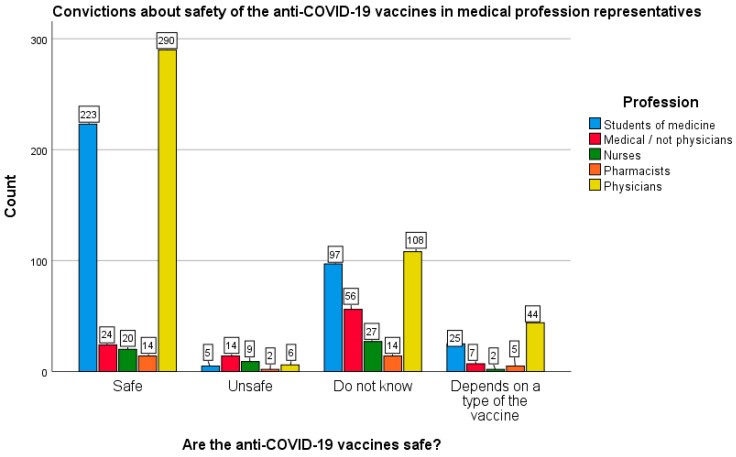
Beliefs about the safety of COVID-19 vaccines among representatives of medical professionals.

On the other hand, in the medical professions group, distrust about vaccine safety was most often declared by other medical personnel (excluding physicians)—55.4%, nurses (46.6%), and pharmacists (40.0%). At the same time, only 24.1% of doctors and 27.7% of medical students expressed similar doubts. In turn, an affirmative answer to the question directly about the harmfulness of vaccines was given by as many as 15.5% of nurses and 13.9% of other medical staff (excluding physicians). On the contrary, only 1.3% of physicians, 1.4% of medical students and 5.7% of pharmacists believed that anti-COVID-19 vaccines could be harmful (χ^2^ (12.992) = 135.527; *p* < 0.005). However, the size of the effect should be assessed as small (Cramer’s V = 0.213; *p* < 0.005). Differences in beliefs about the safety of anti-COVID-19 vaccines among representatives of medical professionals are depicted in Figure 8.

### 3.5. Participating in Discussions about the COVID-19 Vaccinations

Further questions in the survey concerned the talk about anti-COVID-19 vaccines. It has been shown that health professionals more eagerly participated in discussions about COVID-19 vaccines (67.2%) and more often encouraged other people to vaccination (65.8%), as compared with the group of non-medical professions (35.7% and 28.3%, respectively); χ^2^ (2565) = 290.149; *p* < 0.005. The size of the effect is moderate (Cramer’s V = 0.382; *p* < 0.005). The effect persists when gender is considered (women: χ^2^ (2.1424) = 245.019, *p* < 0.005, Cramer’s V = 0.415; *p* < 0.005 vs. men: χ^2^ (3.565) = 58.346, *p* < 0.005, Cramer’s V = 0.321; *p* < 0.005).

Dissuading from vaccination turned out to be rare in the surveyed population. The following difference was found: representatives of non-medical professions significantly more often advised against vaccinating (3.1% of this group) than non-medical students (0.5%), and all respondents related to health care (only 0.2% of them); χ^2^ (16.1976) = 362.758; *p* < 0.005). The size of this effect is small (Cramer’s V = 0.303; *p* < 0.005).

**Figure 9:**
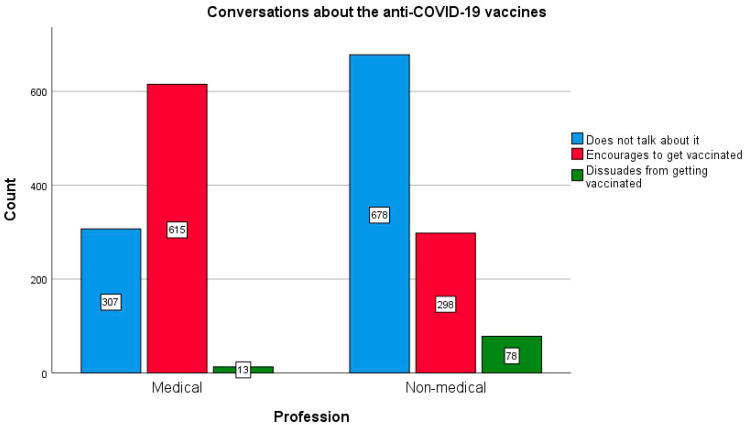
Differences in conversations on the COVID-19 vaccination between medical and non-medical professionals.

**Figure 10:**
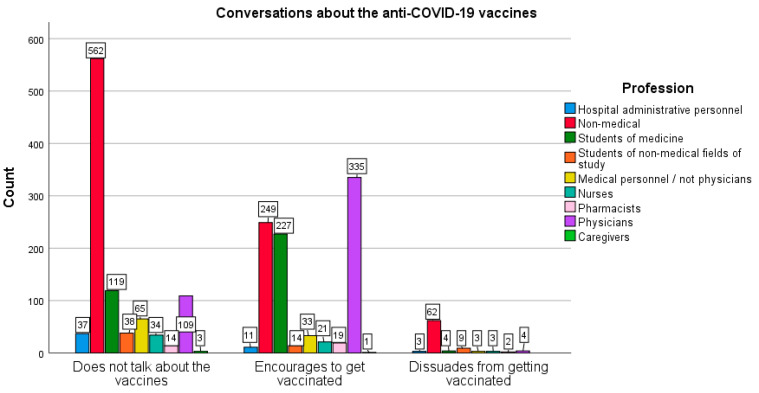
Differences in conversations on the COVID-19 vaccination among the specific medical and non-medical respondents.

Data concerning conversations about vaccinations against COVID-19 undertaken in the two groups of the surveyed population, non-medical and medical professions, is presented in Figure 9 and Figure 10.

### 3.6. Attitudes towards the Mandatory COVID-19 Vaccination

**Figure 11:**
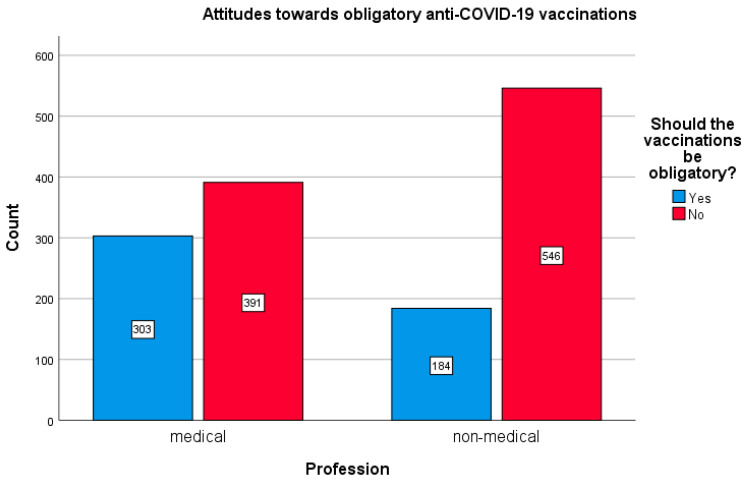
Differences in opinions concerning obligatory COVID-19 vaccination between the medical and non-medical groups.

The attitudes of the respondents towards the issue of mandatory vaccination against COVID-19 were also analyzed. It was revealed that in the non-medical profession group, a significant percentage of the respondents were against mandatory vaccination (72.6%). In the medical groups, there were considerably fewer opponents of mandatory vaccinations (54.5%); χ^2^ (11,989) = 70.036, *p* < 0.005). The size of the effect is small (Cramer’ s V = 0.188; *p* < 0.005). The effect is gender-specific (women: χ^2^ (11,424) = 53.843, *p* < 0.005; Cramer’s V = 0.194; *p* < 0.005 vs. men: χ^2^ (1565) = 19.078; *p <* 0.005; Cramer’s V = 0.184, *p* < 0.005). These opinions of the two compared group out of the surveyed population are shown in Figure 11.

## 4. Discussion

During the COVID-19 pandemic, the possibility of creating an effective vaccine against SARS-CoV-2 seemed to be a desired breakthrough. Vaccinating against COVID-19 is undoubtedly the only manner to control the disease’s dissemination sufficiently under the condition that immunization would be widespread in the population. According to estimations made by Randolph et al. (May 2020), herd immunity is achievable if 67% of the general population is vaccinated [14]. However, the latest prognoses (May 2021) based on an equation estimating the present transmissibility of COVID-19 and the effectiveness of the available vaccines say even up to about 90% of the population should be vaccinated in order to reach herd immunity [15].

A survey conducted on 2–9 June 2020 on a representative sample of 1066 adult Polish citizens (Feleszko et al.) showed that 28% of adults would not plan to be vaccinated against COVID-19 when the vaccine became available. Alarming was that a majority of the reluctant respondents (51%) stated that they would not change their minds even after vaccine safety and efficacy was proved or they were possibly threatened with hefty fines [10]. This data raised some concerns. We assumed that the level of acceptance of vaccines when they were considered only hypothetically should be greater than the declarations made when a new vaccine appeared and the possibility of vaccination with a completely new preparation became real.

In the other paper, published in March 2021 by Szmyd et al., a group of 2300 representatives of the Polish population, of whom 10.96% were physicians and 5.87% administrative healthcare assistants, were interviewed about their attitudes to COVID-19 vaccination. Both above-mentioned groups related professionally to the health care sector demonstrated their willingness to be vaccinated against COVID-19 significantly more often as compared to the control group (82.95% vs. 54.31%, respectively) [16].

The present study was aimed at assessing the attitudes of adult Polish citizens towards COVID-19 vaccination taking into account their relationship with the health care sector. The following groups appeared to express the most positive attitude to the vaccination: doctors (80.6% would like to be vaccinated), medical students (76.9%), and pharmacists (65.7%). Contrary to this, the most skeptical about the prospect of being vaccinated were students of non-medical faculties, of whom only 26.2% declared wanting vaccination and non-medical professions group (38.7% willing), as well administrative-technical staff employed in the healthcare sector (41.2% with positive attitude). Surprisingly, only 43.1% of nurses and 52.1% of the other medical personnel (first responders, physiotherapists, psychologists) declared their willingness to undergo vaccinations in the near future. Differences in vaccine acceptance have been noted between different categories of health care workers both in the present study and in other reports [17,18].

The results obtained are not surprising when it comes to doctors. In the available studies, the percentage of physicians declaring vaccinations against COVID-19 just prior to vaccine’s appearance ranged from 78% to 94.44% [16,18,19,20]. Doctors, obviously, are the most educated and most aware medical group as to validity and safety of vaccinations. Other studies concerning health care workers have also shown that the higher the level of education, the greater the acceptance of vaccinations [8]. Doctors also have a significant amount of direct contact with diseased persons, which puts them at high risk of becoming infected with COVID-19. Perceiving a high risk of infection increases the probability of intention to vaccinate against COVID-19 among health care workers [21].

In addition, a high percentage of medical students surveyed declared their willingness to be vaccinated, which clearly differs from the attitude towards vaccination expressed by students of other majors. For comparison, the other Polish study by Szmyd et al. [22] showed that students of medical and non-medical faculties declared a readiness to take the vaccine for COVID-19 (even 91.99% and 59.42%, respectively), although the percentages were much higher than in our study (76.9% and 26.2%, respectively). On the other hand, in some countries, the percentage of medical students willing to be vaccinated was also not so high, e.g., from 43% in Jordan [23] to 86.1% in Italy [24]. It is worth paying attention to the Italian study, in which students both of medical and non-medical faculties declared comparable high readiness to vaccinate against COVID-19 [24]. It seems very probable that this might be a result of a very dramatic course of the pandemic in this country in 2020. In Poland, the course of the pandemic at the time of our study was much milder. It is commonly known that young people usually perceive themselves as healthy and are not so eagerly involved in pro-health preventive actions as older adults [25]. Medical students who have access to the latest medical knowledge could assume that the COVID-19 vaccination is a chance to bring them back to ‘ordinary life’ and return to the normal course of studies, which undoubtedly influenced their attitudes towards vaccinating. It raises the question as to whether young people are able to consider taking a vaccine in respect to their social aspect, i.e., the risk of infecting more susceptible persons, like older people. In the present study, non-medical students appeared to be the most skeptical group about the COVID-19 vaccination. It is possible that the decisive factors were the low risk of infection or of having a severe course of the disease and lack of medical knowledge. This could also reflect the observed tendency that the desire to be vaccinated increases with age [9,26,27,28,29].

In the available literature, there are few studies assessing pharmacists’ attitudes towards COVID-19 vaccination. In Greek studies, the percentage of acceptance of vaccination among pharmacists was 65%, similar to our study, and also was lower than in the group of doctors [30], whereas in the French study, the relevant percentage was higher at 88.1% [18]. Pharmacists are the professional group that participate in the distribution of vaccines in Poland. Pharmacists act also as patients’ advisors on medicines, hence their opinion about vaccines against COVID-19 may have a large impact on the public perception of vaccination. On the basis of changes in the national vaccination program in Poland, since 30 March 2021, pharmacists have been one of the professional groups allowed to give vaccines against COVID-19, meaning their role in the vaccination action may become much more important.

Considering the acceptance of COVID-19 vaccinations in the surveyed population, our attention was drawn to the negative attitude towards vaccination in the group of nurses. This result seems very surprising. Negative attitudes towards vaccination was declared by nurses despite their medical education and intensive, direct contact with patients, including those with COVID-19, and therefore a high risk of being infected by the coronavirus. What makes this observation even more dangerous is that it is known that nurses in Poland are the professional group which recorded the most cases of COVID-19 and the second largest number of deaths from that virus among healthcare workers (after doctors) [31]. Similar data are observed in other countries. It is worth citing the Israeli research, in which nurses were statistically significantly less likely to accept COVID-19 vaccinations than doctors, and the percentage of nurses who wanted to be vaccinated was even lower than in the general population [19].

This fact should raise concern for two reasons. Firstly, exposure of nurses to the risk of SARS-CoV-2 infection exacerbates personnel shortages in the healthcare system. Secondly, it can be assumed that nurses are likely also unwilling to encourage other people to COVID-19 vaccines, which has a negative impact on public perceptions of this vaccination.

It is known that the nursing profession is very feminized. This fact might affect the attitude towards vaccinations in this professional group. In the present study, men, by contrast to women, turned out to be the group more eagerly declaring the will to be vaccinated. Such gender differences were observed in both groups: medical and non-medical. Men seem to be less anxious about the COVID-19 pandemic [32,33] and more positive as to COVID-19 vaccinations [34]. Another explanation could be the fact that men with COVID-19 are at higher risk for worse outcomes and death [35].

In contrast, women appear to bear the greater psychological cost of the pandemic, and they also express greater concern about vaccination against COVID-19. It is worth emphasizing that women’s skepticism about vaccinations is present both in surveys of the general population and in surveys of healthcare professionals [19,27,28,36]. In contrast with the majority of studies, only individual studies present opposite conclusions, e.g., the previously mentioned study conducted among 19 countries for general population [9] and studies from Saudi Arabia [37] and Ghana [38] both focusing on healthcare workers. Authors of the present paper dare to suggest that such women’s attitude could be due to the fact that women are more often anxious about the future and new experiences, are less prone to risky behaviors or more likely to trust the opinions of ‘pseudo-experts’ and gossips, as compared with men. Obviously, this issue should be for psychologists and sociologists to consider.

In the study by Manning et al., nurses presented unsatisfactory knowledge on the methodology of COVID-19 vaccine production. It is worth emphasizing that the study concerned nursing students and their lecturers [39]. In general, a higher level of knowledge was associated not only with the stronger willingness to be vaccinated but also recommending vaccinations to patients [13,40]. In the survey by Marcu et al., vaccinated HCWs regarded patients’ vaccination as a public health issue and believed that by being vaccinated themselves they could provide a reassuring example to patients, particularly those who have concerns about vaccination [41].

An intriguing aspect of our study was the assessment of the percentage of persons who presented an ambivalent attitude to vaccination against COVID-19, which resulted from some doubts. It was revealed that the percentage of such persons was increasing in the following order: doctors, medical students, pharmacists, non-medical professions, healthcare administration, and students of non-medical faculties. For instance, the percentages of persons still not sure about COVID-19 vaccines was 41.2% in the group of healthcare administrative-technical staff, and more than half (54.1%) among non-medical students. Those persons clearly expressed their expectations for some additional information on the COVID-19 vaccines. As is commonly accepted, unconvinced people should be treated as the main target of experts’ activity if the increase of the percentage of the vaccinated population is planned [36,42].

Further analysis has shown that although more than half of healthcare professionals encouraged people in their environment, both in private and in professional contacts, to take the vaccine, there still remained a significant percentage of this professional group that did not make an attempt to discuss vaccines. What could be the reasons for this lack of desired activity concerning disseminating pro-health awareness? Some factors can be considered: shortage of time, overwork, lack of strong arguments, improper communications, or simply a lack of good will, unfortunately.

What makes things worse is that there was a small group of healthcare professionals who confessed that they discouraged other people from vaccination against COVID-19. This fact is shocking but is not an exception. In the study of Shekhar et al., a large group of healthcare workers who did not intend to take COVID-19 vaccine simultaneously declared that they would not recommend this vaccine to other people [27]. Since healthcare professionals remain the most trusted advisers for the general population [13], negative opinions on vaccination against COVID-19 expressed by healthcare professionals can have a devastating impact on public perception, increase anxiety, and eventually result in strongly discouraging other people from vaccinating.

It was shown that less than 60% of healthcare professionals surveyed by us believed that COVID-19 vaccines were safe, while nearly 30% stated that vaccination was downright unsafe. This latter opinion was probably firmly influenced by the short time from the appearance of newly developed vaccines and the lack of data on their properties and adverse effects among already vaccinated people. Vaccine safety, efficacy and possible side effects seem to be the most important factors determining attitudes towards vaccination against COVID-19 [28,29,36,42].

In this context, the views of the respondents regarding the possibility of introducing obligatory vaccination against COVID-19 seemed interesting. This issue remains controversial in Poland and is currently (November 2021) not considered in the national vaccination program in this country.

In our study, as many as 45.5% of the medical group supported the idea of obligatory vaccinations against COVID-19 and only 27.4% of respondents from the non-medical group. In a December 2020 study from the UAE, 72.9% of healthcare professionals were in favor of compulsory vaccination for all citizens and residents in this country, while 27% were against. In the compulsory vaccination group, respondents were more likely to be vaccinated as soon as a vaccine became available, so probably they are less concerned about side effects [21].

Interestingly, in our study, women, although more skeptical about COVID-19 vaccination, were more likely to recognize the advisability of introducing obligatory vaccinations against this disease. The attitude of surveyed men towards COVID-19 vaccination was more balanced and tolerant: they showed greater acceptance of COVID-19 vaccinations, yet were not in favor of compulsory vaccination.

Many factors influence the attitude towards vaccination. These decisions are based not only on objective facts, but often on subjective beliefs and emotions [3,28,36,42]. It would seem understandable that contact with COVID-19 patients should enhance a will to take a vaccine, which was confirmed in some studies [19], although other authors ascertained that the degree of exposure to the coronavirus did not significantly influence their vaccination decision [28].

People’s approach to voluntary or obligatory vaccination against COVID-19 certainly has a multi-dimensional structure. The safety and effectiveness of vaccines are surely priorities. Other factors should likely also be taken into consideration, like fear of the coronavirus, profession, gender, personality traits, upbringing with a sense of duty or freedom, as well views on civil liberties and the role of the state in citizens’ lives.

For instance, the survey conducted in the US between 30 November and 8 December 2020 by KFF COVID-19 Vaccine Monitor with a sample of 1676 adults showed that among those who were hesitant to get the COVID-19 vaccine, 55% declared a lack of trust in the government’s ability to ensure the vaccines’ safety and effectiveness, 53% showed concerns that the vaccine was too new, and 51% were afraid of the role of politics in the vaccine’s development process. Interestingly, vaccine hesitancy was very high among Republicans (42%) as compared with Democrats (12%) [29].

Attitudes towards COVID-19 vaccination are certainly changing over time [42,43]. In countries with multiple surveys over time, the changes in COVID-19 vaccine acceptance rates were observed. According to the literature review by Salam, in the United Kingdom, the vaccine acceptance rate was 79.0% in April 2020, 83.0% in May 2020, 71.5% in June 2020, 64.0% in July 2020 and 71.7% in September/October 2020 [43]. Until December 2020, opinions of the respondents were rather hypothetical, as they related to vaccines that were just being developed or in clinical trials. Our study presents the social attitude towards COVID-19 vaccination in Poland just before the inauguration of the national vaccination program, in the second half of December 2020. At that time, the public agenda was dominated by concerns about the safety of vaccines created in such a short time and using innovative mRNA technology. The mass media was full of conspiracy theories and fake news about COVID-19 vaccines [44]. Then, in March and April 2021, fears of complications caused by one of the vector vaccines prevailed [45], which also discouraged many people from taking both the first and the second dose of this vaccine.

Towards the end of 2021, willingness to vaccinate remains low in Poland. Starting from November 2021 all persons over 18 years of age could have received the booster dose of a vaccine against COVID-19. Unfortunately, the national vaccination program was generating very little attention, despite the beginning of the fourth wave of the pandemic. Poland is a curious country in the context of attitudes towards the COVID-19 vaccine, because there has not been a large-scale, intensive campaign to promote vaccination, neither during the period when this study was conducted, nor later, when in the summer months of 2021 an interest in vaccinations dropped significantly. At the same time, anti-vaccine views were spreading quite freely in the public space, mainly on the internet and in social networks. The example of many countries, including Italy and France, shows that the creation of benefit mechanisms for vaccinated persons is an effective stimulus to vaccinate. In Poland, there are still no legal solutions which realistically would give more freedom of access to various activities and services to vaccinated people. ‘COVID passports’ are only useful in the case of a trip abroad (situation for November 2021).

As the results of our study showed, in December 2020, conspicuous COVID-19 vaccine hesitancy among some healthcare employees in Poland was a reality. Three months later, according to the data of the Ministry of Health as of 4 April 2021, up to 85% of doctors and 73% of dentists were vaccinated but only 49% of nurses and midwives [46]. Bearing in mind that healthcare professionals were the “zero group” in the Polish vaccination program and that in that time, the third wave of COVID-19 was rising dramatically, these rates of vaccinated persons in the healthcare sector was rather unsatisfactory. Taking into account the good availability of vaccines, even despite some organizing and provision problems, it can be assumed that some healthcare professionals delayed vaccination due to the lack of internal conviction as to the safety and effectiveness of COVID-19 vaccines available at that time.

A slowing rate of vaccination observed in Poland during last few months seems disturbing. The opportunistic attitude of some citizens is just to wait for herd (population) immunity to develop without their own participation. This approach is not only inconsistent with the idea of social solidarity, but also irrational. It should be considered that asymptomatic persons infected with the SARS-CoV-2 are not submitted to isolation, and thus are virus transmitters. Vaccines are not 100% effective and do not protect totally from virus infection. Furthermore, the duration of immunity after vaccination is ambiguous, and some vaccinated people are not able to produce enough resistance to the virus. In Poland, many people against vaccination raise the argument about the right to freedom of choice. The Polish Academy of Sciences comments on this phenomenon with the slogan “There is no freedom without solidarity”, referring to the historical slogan from the times of the fight against communism in Poland [47]. Therefore, in the case of COVID-19, nobody should count only on herd immunity, and the only rational solution is just vaccination of as many members of society as possible [29,48]. In the US, where in June 2021 almost half of the population received at least one dose of the vaccine, still as much as 30% of citizens disclose vaccination hesitancy [29].

In the middle of May 2021, the number of people vaccinated with at least the first dose of the vaccine exceeded 30% of the entire Polish population [49]. According to the survey performed in Poland by Public Opinion Research Center in May 2021, the percentage of respondents declaring a reluctant attitude towards vaccination against COVID-19 was still 25%, although this was less than in January 2021 (30%). It is also disturbing that as many as 40% of people aged 18–29 do not intend to undergo vaccination. People declaring a lack of will to be vaccinated are motivated primarily by fear about the side effects the new vaccines may cause (61%), secondly by doubts as to their low effectiveness (31%), and general reluctance about all vaccinations (18%). Interestingly, relatively few respondents (13%) avoid vaccination because they believe that COVID-19 is not a serious disease [50].

As of 26 August 2021, the number of people vaccinated with at least one dose exceeded 50% of Poles [49]. At the same time, public opinion polls in Poland from that period revealed that only 32% of people who were not yet vaccinated declare that they wanted to use the COVID-19 vaccine. Unfortunately, the percentage of people declaring that there is no such thing that could encourage vaccination reached as much as 43%. The reason people who do not intend to be vaccinated could change their mind was a guarantee of compensation in the event of severe side effects for 35% of respondents, followed by precise information on the frequency of side effects (29% of indications) [51]. The data from October 2021 show that only 53% of Poles are vaccinated with at least one dose, which puts Poland in 23rd place in the European Union [49].

In our interpretation of these data, only a section of unvaccinated people are staunch anti-vaccines. The rest of the population is uninformed about vaccination safety and efficacy and vulnerable to anti-vaccine content that is widely available. In this context, access to information on vaccination, the use of various communication channels and forms of dialogue should be the key elements of the vaccination campaign [1,52,53], more so with a new mutation of SARS-CoV-2, ‘omicron’, becoming increasingly dangerous [54].

### Limitations of the Study

The authors are aware that online surveys are not feasible for accessing the entire population, as their use is limited to people with email, internet and social media access. The inherent coverage bias is a major disadvantage of such an approach. Because of that we could only make valid claims for the particular groups of the survey respondents and were not able to generalize them to the wider population. We decided to use the non-probability sampling mode to collect the data that would be helpful in developing further hypotheses. In our opinion, such an approach is acceptable for exploratory research.

## 5. Conclusions

Understanding the low acceptance of COVID-19 vaccination in the Polish population is crucial for the further course of the pandemic in this country. There are many indications that the virus SARS-CoV-2 will stay with us for a longer period, and its continuous mutations and the dangerous consequences of COVID-19 will probably be a cause for renewing vaccinations each year. Taking this into account, after the first year of the Polish national vaccination program, there is still an urgent necessity to establish an intensive public campaign promoting vaccination against COVID-19 that should be targeting not only the general population but also healthcare providers. The persistence of a disturbingly high percentage of Polish residents reluctant to accept vaccination should pose a challenge for political, administrative and medical authorities to more effectively encourage undecided people to vaccinate against COVID-19.

## Figures and Tables

**Table 1:** Professions of the surveyed participants.

	Number	% of Total	Cumulative %
Administrative and technical hospital personnel	51	2.6	2.6
Non-medical professions	873	44.2	46.8
Students of medicine	350	17.7	64.5
Students of non-medical majors	61	3.1	67.6
Psychologists, rescuers, physiotherapists	100	5.1	72.6
Nurses	54	2.7	75.4
Pharmacists	35	1.8	77.3
Physicians	448	22.7	99.8
Professional caregivers	4	0.2	100.0
Total	1976	100.0	100.0

## Data Availability

The data presented in this study are available on request from the corresponding author upon reasonable request.
